# Systemic T Cell Exhaustion Dynamics Is Linked to Early High Mobility Group Box Protein 1 (HMGB1) Driven Hyper-Inflammation in a Polytrauma Rat Model

**DOI:** 10.3390/cells10071646

**Published:** 2021-06-30

**Authors:** Preeti J. Muire, Martin G. Schwacha, Joseph C. Wenke

**Affiliations:** 1 Combat Wound Care, US Army Institute of Surgical Research, JBSA Ft Sam Houston, San Antonio, TX 78234, USA; joseph.c.wenke.civ@mail.mil; 2 Division of Trauma and Emergency Surgery, Department of Surgery, University of Texas Health Science Center at San Antonio, San Antonio, TX 78229, USA; martin.schwacha@gmail.com

**Keywords:** DAMPs, hyper-inflammation, extremity trauma, lymphopenia, RAGE, TLR4

## Abstract

We previously reported an early surge in high mobility group box protein 1 (HMGB1) levels in a polytrauma (PT) rat model. This study investigates the association of HMGB1 levels in mediating PT associated dysregulated immune responses and its influence on the cellular levels of receptor for advanced glycation end products (RAGE) and toll-like receptor 4 (TLR4). Using the same PT rat model treated with anti-HMGB1 polyclonal antibody, we evaluated changes in circulating inflammatory cytokines, monocytes/macrophages and T cells dynamics and cell surface expression of RAGE and TLR4 at 1, 3, and 7 days post-trauma (dpt) in blood and spleen. Notably, PT rats demonstrating T helper (Th)1 and Th2 cells type early hyper-inflammatory responses also exhibited increased monocyte/macrophage counts and diminished T cell counts in blood and spleen. In blood, expression of RAGE and TLR4 receptors was elevated on CD68^+^ monocyte/macrophages and severely diminished on CD4^+^ and CD8^+^ T cells. Neutralization of HMGB1 significantly decreased CD68^+^ monocyte/macrophage counts and increased CD4^+^ and CD8^+^ T cells, but not γδ^+^TCR T cells in circulation. Most importantly, RAGE and TLR4 expressions were restored on CD4^+^ and CD8^+^ T cells in treated PT rats. Overall, findings suggest that in PT, the HMGB1 surge is responsible for the onset of T cell exhaustion and dysfunction, leading to diminished RAGE and TLR4 surface expression, thereby possibly hindering the proper functioning of T cells.

## 1. Introduction

Polytrauma (PT) patients develop hyper-acute inflammatory responses involving cell death, the release of damage-associated molecular patterns (DAMPs) and immune dysregulation [1,2,3,4,5]. This response impacts patients, especially those with multiple injuries, including fractures, to have adverse clinical outcomes such as altered leukocyte kinetics and impaired fracture repair, in contrast to normal fracture healers [6,7]. Other well-documented clinical consequences of hyper-acute inflammatory responses are systemic inflammatory response syndrome (SIRS) and paralleled by compensatory anti-inflammatory response syndrome (CARS) [8]. PT patients who survive the SIRS-CARS phenomena will experience persistent inflammation, immunosuppression and catabolism syndrome (PICS), rendering them susceptible to opportunistic infections and delayed wound healing [9]. However, the underlying immunological events associated with hyper-inflammation remain elusive. Current evidence suggests that persistent lymphopenia in severe trauma is associated with the development of multiple organ dysfunction syndrome (MODS), impaired fracture healing and increased mortality [5,10]. Thus, this evidence warrants of development of targeted approaches to restore a balanced immune response in PT patients to overcome the burden of deleterious outcomes.

A potential link between sterile trauma-induced immune dysregulation and delayed wound healing are certain pattern recognition receptors that recognize DAMPs such as high mobility group box protein 1 (HMGB1), mitochondrial DNA (mtDNA), S100 and other similar molecules released by dying cells following injury and cell membrane damage [11,12,13]. Despite the importance of all DAMPs in mounting inflammation, here we shed light on the role of HMGB1 in PT due to its prominence as an early post-traumatic predictor marker [14,15]. HMGB1 alerts the immune system and activates inflammatory cascades and cellular chemotaxis [11]. While the onset of inflammation is vital for regulating recovery from trauma and wound healing, if the response is overzealous due to the early burst of HMGB1, the immune cells alter their phenotypes and deprive the wound site of beneficial signals for repair [6,16]. Others and our group have previously reported elevated levels of systemic HMGB1 and altered leukocyte kinetics in PT animal models displaying a delayed fracture healing phenotype [16,17]. Extracellular HMGB1 binds to the receptor for advanced glycation end products (RAGE) and toll-like receptor 4 (TLR4) on immune cells and initiates pro-inflammatory cascades that the host must compensate for to maintain homeostasis [4,18]. Interestingly, the host is equipped with specific innate mechanisms to counter the effect of hyper-immune insults following trauma, which remains unclear and is of interest to our group. However, in extremity trauma, such undertakings by the host’s immune system fail to support adaptive immunological and physiological conditions, leading to deleterious outcomes, thereby requiring therapeutic interventions to modulate mediators to maintain proper functioning. An in vitro study using aortic endothelial cells demonstrated that saturated levels of extracellular HMGB1 lead to cellular exhaustion, causing a negative feedback regulation and ectodomain shedding of RAGE and TLR4 receptors while rendering cells unresponsive to further stimuli [19]. However, these findings lack in vivo validation in a dysregulated inflammatory state. We believe that cellular exhaustion-driven receptor shedding could offer a possible explanation for T cell lymphopenia and dysfunction in PT.

Although the relationship between HMGB1 and immunosuppression has been previously established in single thermal injury models [20,21], whether the early surge in HMGB1 in extremity trauma is implicated as a driving force of hyper-acute inflammation and T cell lymphopenia and dysfunction compared to a single fracture model has not been shown before. Current evidence suggests that peripheral lymphocytes, including certain helper T cell (Th) subtypes, have a role in early immunological responses to thermal injury [22]; however, their responses in the context of PT with burn, blunt trauma and fracture have not been well characterized during the early phase post injuries. We hypothesized that the surge in HMGB1 levels associated with PT dictates the onset of mixed inflammatory responses by altering cellular dynamics and cell surface expression of HMGB1 receptors, RAGE and TLR4, and the early neutralization of HMGB1 will ameliorate such dysregulated immune responses. A previously established PT rat model with a femur osteotomy, blunt chest contusion and scald burn was employed to test this hypothesis [16]. This study’s objective was to shed light on the changes in systemic immune cellular responses, cellular exhaustion/anergy and lymphopenia in PT rats by neutralizing the early systemic HMGB1 levels.

## 2. Materials and Methods

### 2.1. Animals and Surgical Care

All animals were individually housed in ventilated cages and provided unlimited access to food and water and unrestricted activity. Animal procedures were conducted in compliance with the Animal Welfare Act, the implementing Animal Welfare Regulations and the principles of the Guide for the Care and Use of Laboratory Animals, National Research Council. An animal protocol was prepared prior to the study, and the Institutional Animal Care and Use Committee (IACUC) at the United States Army Institute of Surgical Research (USAISR) approved all research conducted in this study. The facility where this research was conducted is fully accredited by the American Association for Accreditation of Laboratory Animal Care (AAALAC). Male Sprague-Dawley rats aged between 10 and 14 weeks and weighing 348–425 g were used in this study. All animals received a pre-surgical dose of buprenorphine-HCL SR (1.2 mg/kg, subcutaneously) at least 15 min before surgery. At surgery, during blood draws and prior to euthanasia, all rats were anesthetized and maintained with 1–3% isoflurane and oxygen delivered via a nose cone on a Bain circuit connected to the rodent gas anesthesia machine (VetEquip Inc., Pleasanton, CA, USA). Naïve rats were not surgically manipulated and served as baseline controls. Post-surgery, rats did not receive any prophylactic antibiotics but were assessed closely for signs of distress and body weight. Rats with ≥10% of body weight loss relative to pre-surgery weight received 3 mL sterile surgical saline subcutaneously. At 1, 3 and 7 days post-trauma (dpt), a volume of ~500 μL of venous blood was collected via a catheter from the tail vein from anesthetized rats. At 7 dpt and immediately after blood collection, the anesthetized rats were humanely euthanized by cardiac exsanguination, and spleens were harvested. Naïve rats were anesthetized and euthanized by cardiac exsanguination, similar to the other rats in this study prior to tissue collection. This study was carried out in compliance with the ARRIVE guidelines [23].

### 2.2. Surgery

Animals were used for the study in two blocks with 5 rats/block. Data from the first block was used to calculate the sample size for the remaining study assuming a power of 80% and an alpha = 0.05, to achieve statistical significance. Rats were divided into two cohorts of trauma representing a single 3 mm femoral osteotomy (OST), i.e., normal fracture repair model and PT, i.e., delayed fracture repair model. The PT rats underwent three traumas, i.e., a 3 mm femoral osteotomy, a blunt chest contusion with a 0.3 kg weight dropped from a height of 68 cm to exert ~2 J of energy on the rat’s chest and a 100 °C water scald burn for 10 s of approximately 20% of the total body surface area. All trauma and surgical procedures were previously described in detail elsewhere [16]. Immediately after post-trauma operations, the OST rats (*n* = 10) and sub-cohorts of PT rats were either left untreated (PT-C; *n* = 10), received a single dose of chicken anti-HMGB1 neutralizing polyclonal antibody (PT-Ab HMGB1; *n* = 10) (Shino-test, Tokyo, Japan; 2 mg/kg, IP) or received single dose of isotype control chicken IgY antibody (PT-IgY; *n* = 5 for 1 and 3 dpt; *n* = 4 for 7 dpt) (Shino-test, Tokyo, Japan; 2 mg/kg, IP). The rats were allowed to recover in clean cages with continued monitoring.

### 2.3. Flow Cytometry

Harvested spleens were weighed, chopped into pieces and gently passed through 70 µm and 40 µm nylon filters with a syringe plunger to prepare single-cell suspensions. Splenocytes from 7 dpt and whole blood from 1, 3 and 7 dpt were subjected to RBC lysis (BioLegend; 1X RBC lysis buffer) and washed with phosphate-buffered saline. Cells were resuspended in FACS buffer (autoMACS rinsing buffer (Miltenyi Biotech) with 2% BSA) and counted by trypan blue exclusion method using the automated cell counter (Countess, Invitrogen). One million cells/sample were stained with a live/dead stain, i.e., zombie violet dye (BioLegend; 1:2000) and anti-rat CD32/Fc block antibody (BD Bioscience; 1:50) before labeling with the fluorescent-labeled detection antibodies. Antibodies used to detect T cells were anti-rat CD3 antibody (viogreen), anti-rat CD4 antibody (PE-Vio770) and anti-rat CD8a antibody (APC-Vio770) (all Miltenyi Biotech, 1:50, 1:10 and 1:10, respectively) and anti-rat γδTCR antibody (PE) (BioLegend; 1:50). Antibodies used to detect CD45^+^ leukocytes and monocyte/macrophage cells were anti-rat CD45 antibody (PE-Cy5) (BD Biosciences; 1:10) and anti-rat CD68 antibody (APC-Vio770) (Miltenyi Biotech; 1:10). Additionally, anti-RAGE antibody (FITC) (Biorbyt; 1:50) and anti-TLR4 antibody (APC) (Novus Biologicals; 1:100) were used to detect surface receptors RAGE and TLR4 on T cells and monocyte/macrophage cells. Cells were labeled for 30 min at 4 °C in the dark and washed twice with FACS buffer. Cells were fixed with fixation buffer (R&D systems) (250 μL/well) for 15 min at 4 °C in the dark and washed twice with FACS buffer before proceeding with data acquisition on the MACS quant 10 flow cytometer (Miltenyi Biotech, Bergisch Gladbach, Germany). All antibodies were titrated before application. Appropriate isotypes control antibodies, fluorescence minus one (FMOs) and single stained cells were used as controls for appropriate gating strategies. Compensation was performed with either single stained cells or beads to ensure there was no spillover within channels. Data were analyzed using Flow Logic software (Miltenyi Biotech), and researchers were blinded to group allocation when analyzing data.

### 2.4. Blood Collection and Processing for Protein Quantification Assays

Aliquots of whole blood from OST (*n* = 5), PT-C (*n* = 5) and PT-Ab HMGB1 (*n* = 5) rats were collected in EDTA tubes and centrifuged at 1000× *g* for 10 min to separate plasma for cytokine analysis. Plasma was stored at −80 °C until used for downstream assays. Cytokines related to Th cell subsets were quantified in the plasma samples using Legend plex rat Th cell cytokine panel kit assay (BioLegend) following the manufacturer’s protocol and plasma dilution of 1:2 for all cytokines, except IL-6 and IFNγ, which was 1:4 dilution. Data were acquired on the MACS quant 10 flow cytometer (Miltenyi Biotech). Data analysis and standard curve interpolation were performed using a BioLegend data analysis software V8.0, supplied with the kit. Additionally, plasma samples were assayed to quantify 67 proteins using a Quantibody^®^ rat cytokine array Q67 kit (RayBiotech quantitative proteomic services). Protein analysis of the plasma was performed by RayBiotech according to their protocol and software analysis. Samples with protein concentrations outside the suggested upper limit of detection and the lower limit of detection were excluded from the study, and the values within the correct detectable range were used for further analysis; hence, we reported cytokine protein results from *n* = 4–5 rats.

### 2.5. Statistical Analysis

Data are reported as mean ± SEM. Statistical analysis was performed using GraphPad Prism version 8.0.0 for Windows, GraphPad Software, San Diego, CA, USA, www.graphpad.com, accessed on 1 June 2021. Data were assessed for normality using the Q-Q plot, homoscedasticity plot, residual plot, D’Agostino and Pearson test, Shapiro–Wilk test, and the difference between means and median. Data that were not normally distributed were log-transformed before statistical analysis. The cytokine array data were assessed by 2-way repeated-measures ANOVA for equal sample numbers and mixed-effect model for samples with missing data with Dunnett’s multiple comparison test. Flow cytometry data from blood was analyzed for statistical significance by performing mixed-effects analysis and Tukey’s Post-hoc multiple comparison test. A *p*-value < 0.05 was deemed significant.

## 3. Results

### 3.1. Pro- and Anti-Inflammatory Cytokines Are Elevated Following Polytraumatic Injuries

**Figure 1:**
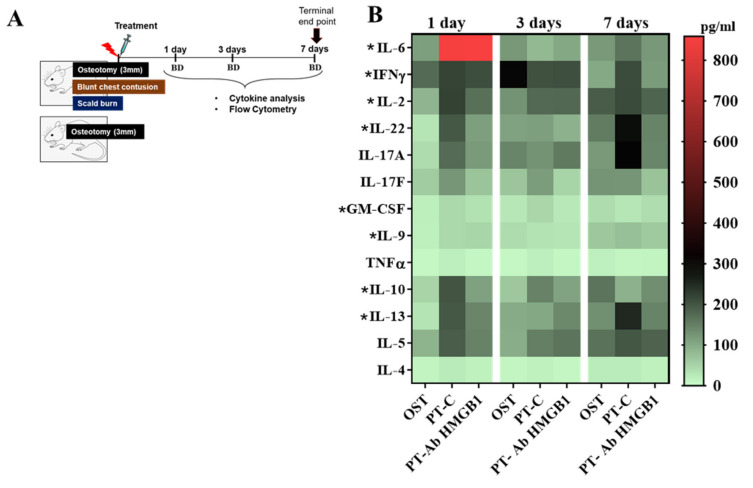
(**A**) Schematic of study design; (**B**) Plasma samples from osteotomy (OST; *n* = 5), polytrauma (PT-C; *n* = 5) and polytrauma + anti-HMGB1 antibody (PT-Ab HMGB1; *n* = 5) at 1, 3 and 7 days post-trauma (dpt) were quantitatively assessed for protein expression of IL-6, IL-17A, IFNγ, IL-13, IL-22, IL-5, IL-2, IL-10, IL17F, IL-9, GM-CSF, IL-4 and TNFα. BD denotes blood draw from the tail vein. * *p* < 0.05 comparing PT-C to OST. The heat map data are presented as median values.

Early inflammatory responses were assessed in circulation from blood drawn from OST, PT-C and PT-Ab HMGB1 rats at 1, 3 and 7 dpt, as depicted in Figure 1A. At 1 dpt, protein levels of T cells secreted cytokines such as interleukin (IL) 22, IL-13, IL-2, IL-6 and Granulocyte-macrophage colony-stimulating factor (GM-CSF) were significantly elevated in PT-C vs. OST (all *p* < 0.05). The anti-inflammatory cytokine IL-10 levels were highest at 1 and 3 dpt, and reduced at 7 dpt in PT-C vs. OST and PT-Ab HMGB1. Cytokines secreted by Th17 cells and γδTCR^+^ T cells such as IL-22, IL-2, IL-17A and IL-17F expression levels were elevated at 1, 3 and 7 dpt in PT-C vs. PT-Ab HMGB1, but were not statistically significant (Figure 1B; Table 1).

### 3.2. Plasma Protein Profiles between PT-C and OST or PT-Ab HMGB1 Rats Are Differentially Expressed

**Figure 2:**
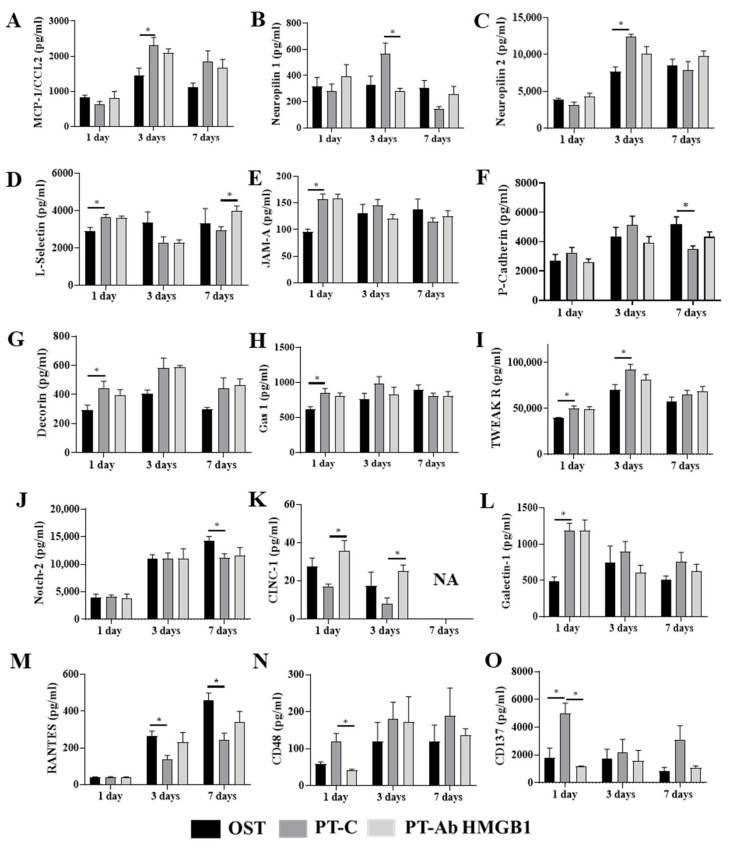
Plasma samples from osteotomy (OST), polytrauma (PT-C), and polytrauma + anti-HMGB1 antibody (PT-Ab HMGB1) at 1, 3 and 7 days post-trauma (dpt) were quantitatively assessed for cytokine protein expression. (**A**) Monocyte attracting chemokine (MCP-1/CCL2); (**B**) Neuropilin 1; (**C**) Neuropilin 2; (**D**–**F**) Adhesion molecules: L-selectin, junctional adhesion molecule (JAM-A) and P-cadherin, respectively; (**G**) Proteoglycan: decorin; (**H**) Cell arrest and apoptosis regulator (GAS-1); (**I**) TNF-related weak inducer of apoptosis receptor (TWEAK R); (**J**) Neurogenic locus notch homolog protein 2 (Notch 2); (**K**) Cytokine-induced neutrophil chemoattractant 1 (CINC-1); (**L**) Anti-inflammatory and T cell suppressive protein: Galectin 1; (**M**) Regulated on activation, normal T cell expressed and secreted (RANTES); (**N**) Lymphocyte activation marker: CD48; and (**O**) lymphocyte activation co-stimulatory immune checkpoint molecule: CD137. (*n* = 4–5 for OST, PT and PT-Ab HMGB1) * *p* < 0.05 comparing OST and PT-Ab HMGB1 rats to PT-C rats. The bar graphs represent the mean, whereas error bars represent SEM. NA–protein expression data is not available.

To better understand the similarities and differences between immune responses in PT-C vs. OST and PT-Ab HMGB1, we examined expression patterns of 67 plasma proteins using the Rat Cytokine Array Q67 (RayBiotech). Fifteen proteins were differentially expressed temporally between PT-C and OST or PT-Ab HMGB1 and are presented in Figure 2. These proteins are Neuropilin 1 and 2, monocyte attracting chemokine (MCP-1/CCL2), adhesion molecules (L-selectin, junctional adhesion molecule (JAM-A), P-cadherin), proteoglycan (decorin), cell arrest and apoptosis regulator (GAS-1), proteins for endothelial differentiation and regeneration of tissue i.e., neurogenic locus notch homolog protein 2 (Notch 2), anti-inflammatory and T cell suppressive protein (Galectin 1), cytokine-induced neutrophil chemoattractant 1 (CINC-1), TNF-related weak inducer of apoptosis receptor (TWEAK R), lymphocyte activation markers, i.e., CD48 and regulated on activation, normal T cell expressed and secreted (RANTES), and lymphocyte activation co-stimulatory immune checkpoint molecule (CD137). Expression levels of proteins responsible for MCP-1/CCL2, neuropilin 1 and neuropilin 2 were elevated in PT-C at 3 dpt compared to OST and PT-Ab HMGB1 rats (all *p* < 0.05) (Figure 2A–C). The expression levels of L-selectin, JAM-A, decorin, galectin-1 and Gas1 increased at 1 dpt in PT-C and PT-Ab HMGB1 compared to OST (all *p* < 0.05); however, L-selectin levels was decreased at 7 dpt in PT-C compared to PT-Ab HMGB1 (*p* = 0.034) (Figure 2D,E,G,H,L). The expression levels of P-Cadherin and Notch-2 were decreased at 7 dpt in PT-C compared to OST (all *p* = 0.04) (Figure 2F,J). The expression levels of CINC-1 were significantly reduced at 1 and 3 dpt in PT-C compared to PT-Ab HMGB1 (*p* = 0.005 and 0.03, respectively) (Figure 2K). In contrast, TWEAK R expression significantly increased at 1 and 3 dpt in PT-C compared to OST (all *p* = 0.04) (Figure 2I). The expression of RANTES decreased in PT-C compared to OST and PT-Ab HMGB1 at 3 and 7 dpt. It was statistically significantly different from OST at 3 and 7 dpt (*p* = 0.013 and 0.005, respectively) (Figure 2M). CD48, a lymphocyte activation marker, increased in PT-C vs. OST and PT-Ab HMGB1 and was statistically significant only on 1 dpt for PT-C vs. PT-Ab HMGB1 (*p* = 0.03) (Figure 2N). Protein expression levels of CD137 increased in PT-C at 1 and 7 dpt and were statistically significant only on 1 dpt for PT-C vs. OST and PT-Ab HMGB1 (*p* = 0.04 and 0.0016, respectively) (Figure 2O).

### 3.3. Neutralization of HMGB1 Alters Circulating Myeloid Cell Counts

**Figure 3:**
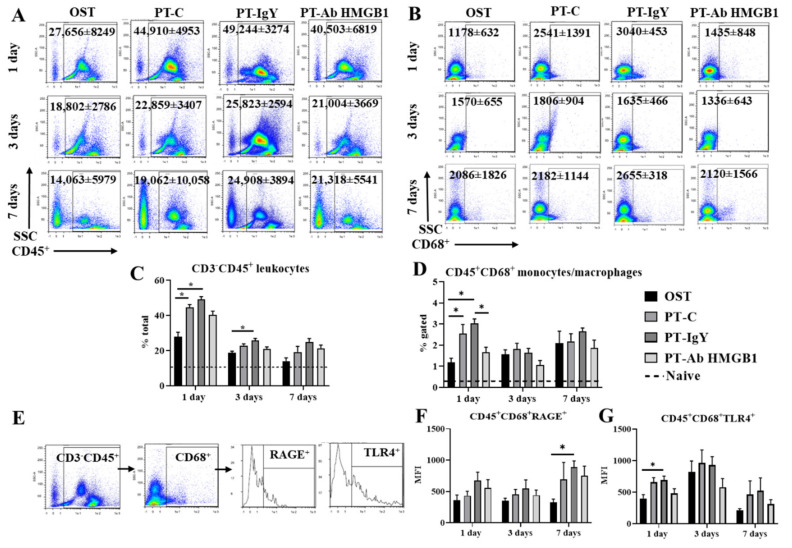
Flow cytometry representative dot plots with mean cell counts ± standard deviation for (**A**) CD45^+^ leukocytes and (**B**) CD68^+^ monocytes/macrophages in blood at 1, 3 and 7 days post-trauma (dpt) in osteotomy (OST; *n* = 10), polytrauma (PT-C; *n* = 10), polytrauma + IgY (PT-IgY; *n* = 5 for 1 and 3 dpt; *n* = 4 for 7 dpt) and polytrauma + anti-HMGB1 antibody (PT-Ab HMGB1; *n* = 10); (**C**) percentage (%) of CD3^−^CD45^+^ leukocytes; (**D**) % CD45^+^CD68^+^ monocyte/macrophages; (**E**) flow cytometry gating scheme to evaluate RAGE and TLR4 surface expression on CD45^+^CD68^+^ monocytes/macrophages; (**F**) Mean fluorescent intensity (MFI) of CD45^+^CD68^+^RAGE^+^ monocytes/macrophages; and (**G**) MFI of CD45^+^CD68^+^TLR4^+^ monocytes/macrophages in blood from rats with OST, PT-C, PT-IgY and PT-Ab HMGB1 at 1, 3 and 7 days. Naïve uninjured rats were used as baseline controls (*n* = 5). The dotted line is the mean of cell counts from naïve rats. * *p* < 0.05 comparing OST, PT-C, PT-IgY and PT-Ab HMGB1 cohorts. The bar graphs represent mean, whereas error bars represent SEM.

To evaluate immune cell counts in PT rats treated with an anti-HMGB1 neutralizing antibody, we performed immunophenotyping of myeloid and lymphoid cells in whole blood 1, 3 and 7 dpt. Immunophenotyping analysis in blood revealed an increased number of CD3^−^CD45^+^ leukocytes at 1 dpt (~37%) and 3 dpt (27%) in PT-C and PT-IgY vs. OST (all *p* < 0.05) (Figure 3C). CD45^+^CD68^+^ monocytes/macrophages were significantly increased at 1 dpt (~61%) in PT-C and PT-IgY vs. OST (*p* = 0.04 and 0.0003, respectively) (Figure 3D). The mean fluorescence intensity (MFI) of RAGE expression on CD45^+^CD68^+^ cells was significantly elevated in PT-IgY vs. OST at 7 dpt (*p* = 0.002) (Figure 3F), and MFI of TLR4 expression on CD45^+^CD68^+^ cells was significantly increased in PT-IgY vs. OST at 1 dpt (*p* = 0.02) (Figure 3G). Following anti-HMGB1 antibody treatment, the PT-Ab HMGB1 rats displayed no statistically significant differences in the levels of circulating CD3^−^CD45^+^ leukocytes, CD45^+^CD68^+^RAGE^+^ cells and CD45^+^CD68^+^TLR4^+^ cells when compared to PT-C or PT-IgY rats (Figure 3C, Figure 3F and Figure 3G, respectively); however, there was an average of a 45% decrease in CD45^+^CD68^+^ monocyte/macrophage counts in PT-Ab HMGB1 vs. PT-IgY rats (Figure 3D).

### 3.4. Neutralization of HMGB1 Improves Circulating CD4^+^T and CD8^+^T Cell Counts

**Figure 4:**
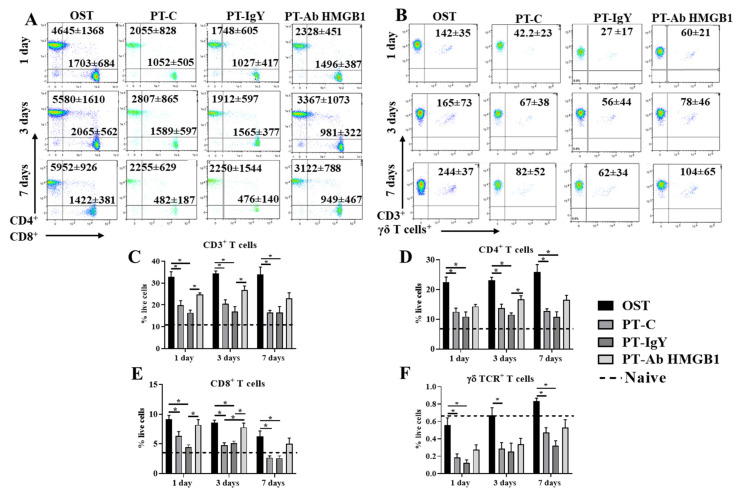
Flow cytometry representative dot plots with mean cell counts ± standard deviation for (**A**) CD3^+^CD4^+^ and CD3^+^CD8^+^ T cells and (**B**) CD3^+^γδTCR^+^ T cell counts in blood at 1, 3 and 7 days post-trauma (dpt) in osteotomy (OST; *n* = 10), polytrauma (PT-C; *n* = 10), polytrauma + IgY (PT-IgY; *n* = 5 for 1 and 3 dpt; *n* = 4 for 7 dpt) and polytrauma + anti-HMGB1 antibody (PT-Ab HMGB1; *n* = 10) rats; (**C**) percent (%) CD3^+^; (**D**) % CD3^+^CD4^+^; (**E**) % CD3^+^CD8^+^; (**F**) % CD3^+^γδTCR T cells in blood from rats with OST, PT-C, PT-IgY and PT-Ab HMGB1 at 1, 3 and 7 dpt. Naïve uninjured rats were used as baseline controls (*n* = 5). The dotted line is the mean of cell counts from naïve rats. * *p* < 0.05 comparing OST, PT-C, PT-IgY and PT-Ab HMGB1 cohorts. The bar graphs represent mean, whereas error bars represent SEM.

We determined CD3^+^CD4^+^ helper, CD3^+^CD8^+^ cytotoxic and CD3^+^γδTCR^+^ T cell counts at 1, 3 and 7 dpt in OST, PT-C, PT-IgY and PT-Ab HMGB1. Flow cytometry results suggest a 40–50% decrease in CD3^+^, CD3^+^CD4^+^, CD3^+^CD8^+^ and CD3^+^γδTCR^+^ T cell counts across 7 dpt in PT-C compared to OST (all *p* < 0.05) (Figure 4C–F). Interestingly, following the neutralization of HMGB1, there was increased blood CD3^+^, CD3^+^CD4^+^ and CD3^+^CD8^+^ T cell counts. PT-Ab HMGB1 rats demonstrated a statistically significant increase of CD3^+^CD4^+^ T cell counts at 3 dpt (30%) (*p* = 0.021) (Figure 4D) and CD3^+^CD8^+^ T cells counts on 1 dpt (45%) and 3 dpt (39%) (*p* = 0.008 and 0.022, respectively) (Figure 4E) compared to PT-C or PT-IgY; however, the counts were only moderately increased and were not as high as observed in the OST rats. The levels of circulating γδ^+^TCR T cell counts were modestly increased (10–20%) in PT-Ab HMGB1 rats compared to PT-C and PT-IgY rats but were not statistically significant (Figure 4F). As hypothesized, the neutralization of HMGB1 immediately post-injuries ameliorates CD3^+^CD4^+^ and CD3^+^CD8^+^ T cell suppression in circulation.

### 3.5. Neutralization of HMGB1 Restores RAGE and TLR4 Surface Expression on Circulating T Cells

**Figure 5:**
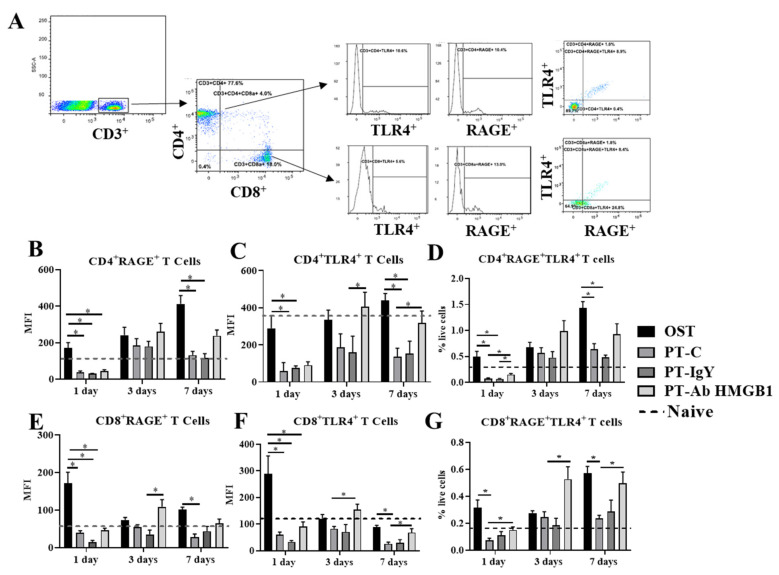
(**A**) Flow cytometry gating scheme to quantify CD4^+^ and CD8^+^ T cells expressing RAGE and TLR4 surface receptors in blood at 1, 3 and 7 days post-trauma (dpt) in osteotomy (OST; *n* = 10), polytrauma (PT-C; *n* = 10), polytrauma + IgY (PT-IgY; *n* = 5 for 1 and 3 dpt; *n* = 4 for 7 dpt) and polytrauma + anti-HMGB1 antibody (PT-Ab HMGB1; *n* = 10) rats; (**B**) mean fluorescence intensity (MFI) of CD4^+^RAGE^+^ T cells; (**C**) MFI of CD4^+^TLR4^+^ T cells; (**D**) percent (%) of CD4^+^RAGE^+^TLR4^+^ T cells in blood; (**E**) MFI of CD8^+^RAGE^+^ T cells; (**F**) MFI of CD8^+^TLR4^+^ T cells; (**G**) % of CD8^+^RAGE+TLR4+ T cells in blood. Naïve uninjured rats were used as baseline controls (*n* = 5). The dotted line is an average of cell counts from naïve rats. * *p* < 0.05 comparing OST, PT-C, PT-IgY and PT-Ab HMGB1 cohorts. The bar graphs represent mean, whereas error bars represent SEM.

To investigate the biological effects of HMGB1 on T cells’ functional status and their receptor expression, we evaluated the surface expression of RAGE and TLR4 expression on circulating CD3^+^CD4^+^ and CD3^+^CD8^+^ T cells at 1, 3 and 7 dpt. The expression of RAGE and TLR4 on CD3^+^CD4^+^ T cells and CD3^+^CD8^+^ T cells was significantly lower in the PT-C rats compared to OST rats at 1 and 7 dpt (*p* < 0.05) but not at 3 dpt (*p* > 0.05) (Figure 5B, C, E and F). Following treatment with the anti-HMGB1 antibody, the CD3^+^CD4^+^ T cells had increased TLR4 expression in PT-Ab HMGB1 rats compared to PT-C or PT-IgY rats at 3 and 7 dpt (*p* = 0.047 and 0.035, respectively) (Figure 5C). CD4^+^ cells co-expressing RAGE and TLR4 significantly increased in PT-Ab HMGB1 rats compared to PT-C and PT-IgY rats at 1 dpt (*p* = 0.037 and 0.017, respectively) but the increase was not statistically significant at 3 and 7 dpt (*p* > 0.05) (Figure 5D). CD3^+^CD8^+^ T cells increased RAGE expression in PT-Ab HMGB1 rats compared to PT-IgY rats at 3 dpt (*p* = 0.012) (Figure 5E); CD3^+^CD8^+^ T cells also increased TLR4 expression at 3 and 7 dpt (*p* = 0.014 and 0.042) (Figure 5F). CD3^+^CD8^+^ cells co-expressing RAGE and TLR4 significantly increased in PT-Ab HMGB1 rats compared to PT-C and PT-IgY rats across 7 dpt (all *p* < 0.05) (Figure 5G).

### 3.6. Neutralization of HMGB1 Does Not Affect Splenic T Cell Counts

**Figure 6:**
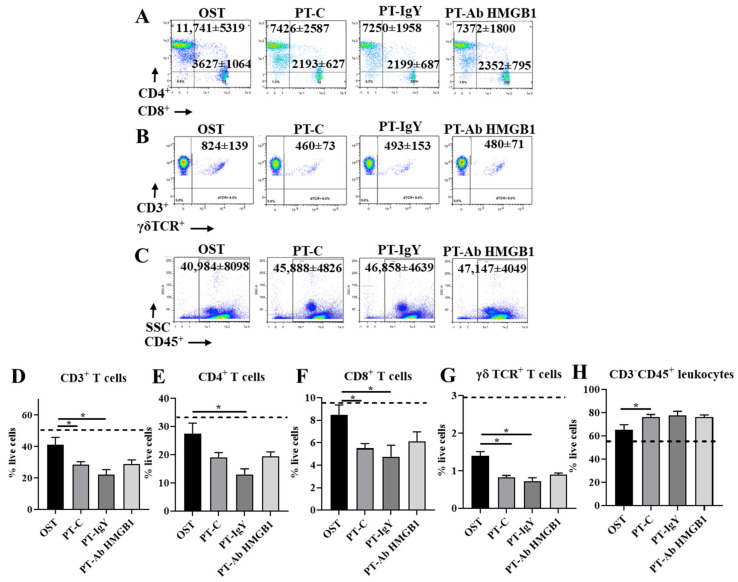
Flow cytometry representative dot plots with mean cell counts ± standard deviation for (**A**) CD3^+^CD4^+^ and CD3^+^CD8^+^ T cells; (**B**) CD3^+^γδTCR^+^ T cell counts; (**C**) CD3^−^CD45^+^ leukocytes in blood at 1, 3 and 7 days post-trauma (dpt) in osteotomy (OST; *n* = 10), polytrauma (PT-C; *n* = 10), polytrauma + IgY (PT-IgY; *n* = 5 for 1 and 3 dpt; *n* = 4 for 7 dpt) and polytrauma + anti-HMGB1 antibody (PT-Ab HMGB1; *n* = 10) rats; (**D**) percentage (%) CD3^+^ T cells; (**E**) % CD3^+^CD4^+^ T cells; (**F**) % CD3^+^CD8^+^ T cells; (**G**) % CD3^+^γδTCR T cells; and (**H**) % CD3^−^CD45^+^ leukocytes in blood from rats with OST, PT-C, PT-IgY and PT-Ab HMGB1 at 1, 3 and 7 dpt. Naïve uninjured rats were used as baseline controls (*n* = 5). The dotted line is the mean of cell counts from naïve rats. * *p* < 0.05 comparing OST, PT-C, PT-IgY and PT-Ab HMGB1 cohorts. The bar graphs represent mean, whereas error bars represent SEM.

At 7 dpt, splenocytes were evaluated for the immune-modulating role of HMGB1 on myeloid and lymphoid cell dynamics. Similar to myeloid and lymphoid cells in the blood, the splenic CD3^+^, CD3^+^CD4^+^, CD3^+^CD8^+^ and CD3^+^γδTCR^+^ T cells decreased (60–70%) (Figure 6D–G) and CD3^−^CD45^+^ leukocytes increased (~10%) (Figure 6H) at 7 dpt in PT-C compared to OST (all *p* < 0.05). However, treatment with anti-HMGB1 antibody did not alter circulating CD3^+^ T cells and CD3^−^CD45^+^ leukocytes compared to non-treated PT rats, i.e., PT-C and PT-IgY (Figure 6D–H).

## 4. Discussion

**Figure 7:**
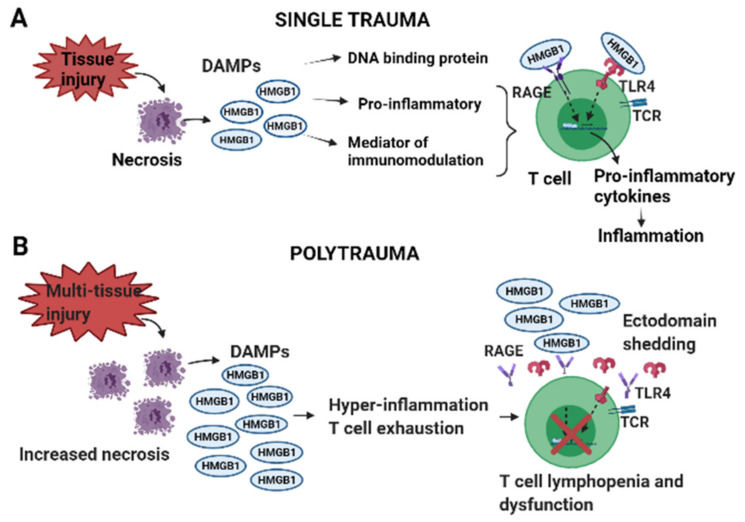
Graphical illustration showing the effects of high mobility group box protein 1(HMGB1), a damage-associated molecular pattern (DAMP) in (**A**) single trauma and (**B**) polytrauma (PT). Several DAMPs such as HMGB1, mitochondrial DNA (mtDNA), S100 and other similar molecules are released from the cytosol of necrotic cells following injury and the damage of the cell membranes. Despite the importance of all DAMPs in mounting inflammation, here we shed light on the role of HMGB1 in PT due to its prominence as an early post-traumatic predictor marker [14,15]. Following injury circulating levels of HMGB1 are relatively lower in a single injury than in PT. HMGB1 mediates pro-inflammatory and immunomodulatory responses by binding to receptor for advanced glycation end products (RAGE) and toll-like receptor 4 (TLR4) on immune cells and triggers pro-inflammatory cascades in the surrounding cells the host must compensate for to maintain homeostasis. However, while inflammation is vital for regulating tissue homeostasis and repair if overburdened by the early surge of HMGB1, it can result in hyper-inflammation and immune cell exhaustion causing T cells to alter their phenotypes and undergo ectodomain shedding of RAGE and TLR4, leading to T cell lymphopenia and dysfunction.

An established rat PT model of delayed fracture healing exhibiting HMGB1 surge was employed in this study. To investigate the previously unknown mechanisms between HMGB1 and dysregulated immune responses, mainly T cell exhaustion, lymphopenia and dysfunction in PT, we determined whether PT associated HMGB1 levels dictate the onset of early mixed inflammatory responses, including altered cellular expression of RAGE and TLR4 and if neutralization of HMGB1 ameliorates such dysregulated immune responses. Our rat PT model exhibited massive and complex early hyper-inflammatory responses as indicated by the elevated levels of cytokines, chemokines, adhesion molecules, T cell activation, as well as T cell suppressive cytokines, cell arrest and apoptosis proteins. Further, there was a concurrent rise in monocyte/macrophages and severely diminished T cells in the blood and spleen. While treatment with anti-HMGB1 antibody immediately post-trauma modestly attenuated the early hyper-inflammatory cytokine responses, it significantly restored immune cell dynamics and receptor expression of RAGE and TLR4 in blood, but not in the spleen. Such interactions could be potentiating cellular hyper-activation and exhaustion, rendering cells unresponsive to further stimuli, a possible reason for the delayed fracture healing phenotype associated with PT (Figure 7).

During the initial days post-trauma, our observations of the immune responses conform to expectations based on the previous clinical observations and those observations made in other rodent models [20,21]. Studies using rodent models of trauma-hemorrhage and sepsis have demonstrated similar concurrent responses showing both hyper-inflammation and immunosuppression, thereby establishing an independent response pattern known as mixed antagonist response syndrome (MARS) that, according to our results, seems to be also prevalent in PT [24,25,26]. The rise in plasma concentrations of cytokines such as IL-6, IFNγ and TNFα, during the early hours post-trauma (hpt) likely contributes to the mobilization of CD45^+^ myeloid leukocytes, including CD68^+^ monocyte/macrophages. Meanwhile, elevated levels of GM-CSF and MCP-1 observed here may associate with migration and proliferation of monocyte/macrophages within the inflammatory niche, as previously demonstrated [27]. As observed in severe trauma patients, a notable SIRS response, paralleled by CARS-like responses associated with elevated anti-inflammatory molecules such as IL-10, IL-13 and Galectin, was evident in our results, confirming a MARS-like response in PT [28]. The comparatively lower TNFα expression coincides with higher IL-10 levels in the current study, which is known to inhibit TNFα secretion. Schneider et al. showed elevated levels of IL-10, IL-6 and TNFα during the early trauma hemorrhage and blocking of IL-10 further increased IL-6 and TNFα levels after injury, indicating that IL-10 release may be part of the body’s natural defense process to counter-regulate the early-deranged immune mediators as means to attenuate post-injury SIRS [25]. This observation could explain the reason for increased IL-6 expression at 1 dpt in PT-Ab HMGB1 rats, whose IL-10 levels were slightly reduced during that time-point, compared to PT-C rats.

Contrasting to the increased proportion of monocyte/macrophages in blood and spleen, the T cell counts were significantly depleted, consistent with previous findings suggesting trauma-associated lymphopenia in trauma patients [5]. As suspected, early and inappropriate T cell activation was confirmed with increased expression of lymphocyte activation markers, CD48 and CD137, at 1 dpt in PT-C compared to OST and PT-Ab HMGB1. Cross-linking of CD137 promotes IL-2 secretion and enhances T cell proliferation, survival and cytolytic activity, a possible explanation for increased IL-2 levels in PT-C than OST and PT-Ab HMGB1 at 1 dpt. Interestingly and contrasting to previous findings, the expression of RANTES, a chemoattractant for T cells, decreased in PT-C compared to PT-Ab HMGB1 and OST at 3 and 7 dpt, which is indicative of T cell depletion in PT-C [24]. The role of HMGB1 in activating CD4^+^ T cells has been previously demonstrated [29]; however, in the context of extremity trauma, the untimely activation of T cells via HMGB1 mediated cascades within the first 24 hpt could explain the occurrence of T cell exhaustion and dysfunction. Early neutralization of HMGB1 significantly ameliorated circulating CD4^+^ and CD8^+^ T cell depletion. Interestingly, the same anti-HMGB1 antibody treated PT rats demonstrated no significant recovery of γδTCR^+^ T cells, suggesting the potential involvement of additional mediators in regulating γδTCR^+^ T cells depletion in PT, prompting further investigation.

Recent findings in chronic inflammatory disorders and cancer suggest that HMGB1 enhances immunosuppressive properties of T cells either directly or indirectly [30]. Direct activation occurs via RAGE and TLR4 surface receptors expressed on T cells. For example, the HMGB1:RAGE/TLR4 axis mediates activation of immune inhibitory function of regulatory T cells (Tregs), whose primary role is to suppress CD4^+^ and CD8^+^ T cells via the production of IL-10 [30]. Whereas indirect activation could be via HMGB1:TLR4: NFκB axis mediated activation of myeloid-derived suppressor cells (MDSCs) proliferation and its crosstalk with macrophages resulting in enhanced IL-10 secretion, could potentially exert T cell suppression [31]. The exact mechanisms of T cell suppression are still not fully understood and could be a combination of both direct and indirect mechanisms involving RAGE and TLR4 receptors as well as via MDSCs, which has been previously described [20]. While the expression levels of RAGE and TLR4 increased on monocytes/macrophages, the receptor expression levels significantly diminished on T cells in PT-C compared to OST. This study is the first indication of diminished levels of surface expression of RAGE and TLR4 on T cells in response to overwhelming levels of HMGB1 in vivo. In an in vitro study, the authors stimulated human aortic endothelial cells (HAECs) with a high concentration of HMGB1 and demonstrated that as time progressed, HAECs rapidly underwent ectodomain shedding of RAGE and TLR4 in response to HMGB1 and was concentration and time-dependent, which caused the cells to become insensitive to further HMGB1 stimulation [19]. Similarly, in our study, the diminished expression levels of RAGE and TLR4 on T cells in PT could be due to receptor ectodomain shedding and T cell exhaustion/anergy because of the early and untimely activation of T cells by HMGB1. Interestingly, early neutralization of HMGB1 restored the expression of RAGE and TLR4 on T cells (primarily CD8^+^ T cells), implying that HMGB1 favors receptor-mediated immune function when present at low levels, thereby suggesting its direct role in regulating T cell responses via RAGE and TLR4. Further, the proportion of T cells expressing RAGE and TLR4 increased, accompanied by the augmentation of cell-cell adhesion ability evidenced by elevated plasma levels of L-selectin and P-cadherin, which are rapidly shed by specialized enzymes upon cellular activation, suggesting that neutralization of HMGB1 demonstrated a positive response for maintaining the CD4^+^ and CD8^+^ T cells activation in blood. We and others have shown that circulating L-selectin levels are increased at 1 dpt in PT-C, and the levels continue to decrease with time in extremity trauma [32]. Importantly, our observations likely indicate that the use of therapeutic blocking agents specific to RAGE or TLR4 for long-term suppression of HMGB1 mediated inflammatory pathways might not deliver favorable outcomes in PT.

In addition to evaluating T cell counts by flow cytometry, we also attempted to characterize the helper T (Th) cell responses in PT based on the Th cell cytokine profiles in circulation. Recent findings support the notion that the induction of specific Th cell subsets are critical for orchestrating the immune regulatory mechanisms and have emerged as essential reparative cells in wound healing. T cells can resolve inflammation by secreting reparative cytokines and growth factors and interact with other immune cells to potentiate the complex and active tissue repair process (reviewed in [33]). Some trauma studies suggest that an abnormal shift within the Th cell subsets and their secreted cytokines contributes to dysregulated immune responses [22,34]. However, this theory remains unclear due to contrasting findings from various research groups. In the case of trauma hemorrhage patients, a study demonstrated that Th type 1 cell (Th1) responses (secrete IL-2, IFNγ) were suppressed. In contrast, the Th type 2 (Th2) responses (secrete IL-4, IL-5, IL-13 and IL-10) were elevated, leading to a suppressed adaptive immune response and increased susceptibility to sepsis [35]. Findings from another group indicate that following severe burn trauma, a notable increase in Th2 response occurs without altering the Th1 response [36,37]. In the current study, we assessed circulating Th cytokines secreted from various Th cell subtypes and showed that most of the Th cytokines (with either pro-inflammatory or anti-inflammatory roles) peaked on day one in PT-C compared to OST rats. The elevated levels of IL-22, IL-13, IL-2 and IL-6 observed within the first 24 hpt may likely provide integral signals for T cell activation and mobilization. During the first 24 h, the cytokines released do not indicate a favorable shift in the Th1/Th2-type towards Th2 cytokine response. However, cytokine data suggests that more than one of the Th cell subtypes were simultaneously activated. These observations are similar to those demonstrated previously in major trauma patients with generally high plasma levels of all Th cell cytokines, who needed hospitalization but did not succumb to trauma [28].

As mentioned previously, the increased cytokine levels during the initial 24 hpt depict the occurrence of a cytokine storm, which is of diagnostic value in severe trauma patients with a risk of developing severe complications and death [38]. We suspect that a prominent Th2 immunosuppressive response is marked by elevated IL-10 levels, indicating post-injury T cell suppression in PT. Shimin Wang et al., in their study, demonstrated that increased IL-10 production led to T cell inactivation and impairment of adaptive immunity in the tumor environment [39]. IL-10 is also known to suppress the production of pro-inflammatory mediators such as IL-2 and IFNγ secreted by Th1 cells, thereby inhibiting innate and adaptive immune responses to infectious pathogens. IL-2 is predominantly expressed by activated CD4^+^ T cells, and its levels are generally quantified to assess Th1 responses. Inadequate IL-2 production is associated with immunosuppression after burn injury [40]. Contrastingly, we observed elevated levels of IL-2 in PT-C compared to OST at 1 dpt and diminished levels at 3 and 7 dpt in all groups. High levels of IL-2 in our study are per the findings of Petr Svoboda et al., where the authors reported that IL-2 was detected in severe trauma patients only during the first 24 hpt, following which the concentrations of IL-2 rapidly decreased in all trauma patients [41]. Pro-inflammatory cytokines such as IL-22, IL-2, IL-17A and IL-17F secreted by Th17 cells and γδTCR^+^ T cells were substantially elevated at all times in PT-C vs. PT-Ab HMGB1, indicating the onset of an early hyper-inflammatory response. However, whether this early hyper-inflammatory response in PT is due to a shift in precisely the Th2 response or could be due to global immune depression is unknown and requires further evaluation of the Th cell subtypes. Our findings infer that the heightened cytokine response is the body’s natural way of regulating the immune response. If left in such an overwhelming inflammatory state, the response could have deleterious effects, leading to MODS and delayed healing.

Overall, these findings contribute to the characterization of polytraumatic peripheral hyper-inflammation and T cell exhaustion, lymphopenia and dysfunction. We suggest that modulating the balance between the pro-and anti-inflammatory mediators in a timely fashion may become a potential therapeutic approach that reduces mortality and improve the prognosis of PT patients. There are a few limitations to the study. Since the animals were raised in an almost sterile environment, the phenotype of the CD8^+^ T cells may differ from clinical data, especially those trauma patients who have been previously exposed to various antigens creating an individualized immune profile [42], raising the notion that further evaluation of memory CD8^+^ T cells in this model is desirable for reaching definitive conclusions. Although the distribution of investigated circulating Th cytokines levels indicated no particular shift toward a specific Th cell subset, the Th cell subset distribution may differ in the tissue environment and need further investigation. Another limitation is that the cytokines secreted by Th cells may also be secreted by other leukocytes, which could be interfering with clearly distinguishing a particular shift toward a specific Th cell subset.

## 5. Conclusions

In conclusion, our results indicate that T cell exhaustion and lymphopenia are possibly associated with the altered expression levels of RAGE and TLR4, which is linked to the rise in leukocyte mobilization and an early mixed hyper-inflammatory response by a surge in HMGB1 levels following extremity trauma. We ultimately demonstrated that high levels of HMGB1 are responsible for producing unsatisfactory outcomes in PT; nevertheless, its early neutralization immediately post injuries opens up new opportunities for early and targeted therapeutic strategies for preventing dysregulated immune responses after polytraumatic injuries.

## Figures and Tables

**Table 1:** Mean difference and 95% confidence interval (CI) of cytokine protein concentrations (pg/mL) in plasma samples from osteotomy (OST; *n* = 5), polytrauma (PT-C; *n* = 5) and polytrauma + anti-HMGB1 antibody rats (PT-Ab HMGB1; *n* = 5) at 1, 3 and 7 days post-trauma (dpt). * *p* < 0.05 comparing OST and PT-Ab HMGB1 rats to PT-C rats.

			1 dpt		3 dpt		7 dpt	
Cytokines	Pro- /Anti- Inflammatory Role	Helper/ Regulatory T Cell (Th/Treg) Cell Type	PT-C vs. OST	PT-C vs. PT-Ab HMGB1	PT-C vs. OST	PT-C vs. PT-Ab HMGB1	PT-C Vs. OST	PT-C vs. PT-Ab HMGB1
IL-6	Pro- inflammatory	Th1, Th2, Th17 and Th22	813 (8 to 162) *	−47 (−919 to 825)	−68 (−162 to 26)	23 (−64.33 to 109.4)	66 (−215 to 346)	25 (−253 to 303)
IL-17A	Pro- inflammatory	Th17	105 (−100 to 310)	60 (−57 to 178)	44 (−205 to 294)	−14 (−352 to 323)	70 (−114 to 254)	43 (−145 to 231)
IFNγ	Pro- inflammatory	Th1 and Th17	0.14 (−0.6 to 0.9)	−0.03 (−0.7 to 0.63)	−0.28 (−0.8 to 0.3)	0.01 (−0.4 to 0.4)	0.6 (0.02 to 1.2) *	−0.003 (−1.04 to 1.03)
IL-22	Pro- inflammatory	Th17 and Th22	150 (38 to 262) *	67 (−53 to 187)	1.2 (−83 to 85)	−104 (−432 to 223)	123 (−96 to 341)	110 (−108 to 328)
IL-2	Pro- inflammatory	Th1	137 (28 to 246) *	36 (−82 to 153)	45 (−124 to 213)	1.6 (−172 to 175)	14 (−165 to 193)	58 (−102 to 218)
IL-17F	Pro- inflammatory	Th17	60 (−16 to 136)	35 (−42 to 112)	43 (−115 to 200)	36 (−94 to 165)	12 (−77 to 101)	38 (−36 to 113)
IL-9	Pro- inflammatory	Th9	0.9 (0.2 to 1.7)	0.2 (−0.8 to 1.2)	0.14 (−1.2 to 1.5)	−0.3 (−1.9 to 1.3)	−0.02 (−1.3 to 1.3)	−0.006 (−1.3 to 1.3)
GM-CSF	Pro- inflammatory	Th1, Th2 and Th17	0.9 (0.01 to 1.7)	0.3 (−0.43 to 1.04)	0.4 (−1.04 to 1.8)	0.3 (−1.14 to 1.7)	0.06 (−1.3 to 1.4)	−0.2 (−1.6 to 1.3)
TNFα	Pro- inflammatory	Th1, Th2, Th17, Th22 and Th9	11 (−1.2 to 23)	4 (−19 to 27)	6 (−25 to 37)	6 (−22 to 34)	−1.03 (−14 to 12)	0.3 (−11 to 12)
IL-10	Anti- inflammatory	Th1, Th2, Tregs, Th17, Th9 and Th22	116 (33 to 199) *	20 (−119 to 158)	63 (−67 to 192)	49 (−81 to 178)	−35 (−161 to 91)	−97 (−410 to 217)
IL-13	Anti- inflammatory	Th2 and Th22	131 (6 to 256) *	59 (−64 to 181)	37 (−174 to 248)	−34 (−344 to 277)	66.14 (−95 to 228)	42.01 (−126 to 210)
IL-5	Anti- inflammatory	Th2	98 (−53 to 248)	43 (−110 to 195)	61 (−82 to 204)	2.4 (−171 to 175)	−1.03 (−144 to 142)	4.3 (−127 to 136)
IL-4	Anti- inflammatory	Th2	8.7 (−6 to 23)	3 (−13 to 20)	6 (−16 to 28)	5 (−14 to 23)	−0.6 (−18 to 17)	2 (−18 to 22)

## Data Availability

The datasets generated during and/or analyzed during the current study are available from the corresponding author on reasonable request.

## References

[1] Karwan K. (2009). Evaluation of patients with polytrauma treated in the emergency department. Pol. Merkur. Lek. Organ Pol. Tow. Lek..

[2] Dhar S.A., Butt M.F., Hussain A., Mir M.R., Halwai M.A., Kawoosa A.A. (2008). Management of lower limb fractures in polytrauma patients with delayed referral in a mass disaster: The role of the Ilizarov method in conversion osteosynthesis. Injury.

[3] Hranjec T., Swenson B.R., Dossett L.A., Metzger R., Flohr T.R., Popovsky K.A., Bonatti H.J., May A.K., Sawyer R.G. (2010). Diagnosis-dependent relationships between cytokine levels and survival in patients admitted for surgical critical care. J. Am. Coll. Surg..

[4] Xiao W., Mindrinos M.N., Seok J., Cuschieri J., Cuenca A.G., Gao H., Hayden D.L., Hennessy L., Moore E.E., Minei J.P. (2011). A genomic storm in critically injured humans. J. Exp. Med..

[5] Manson J., Cole E., De’Ath H.D., Vulliamy P., Meier U., Pennington D., Brohi K. (2016). Early changes within the lymphocyte population are associated with the development of multiple organ dysfunction syndrome in trauma patients. Crit. Care.

[6] Weckbach S., Hohmann C., Braumueller S., Denk S., Klohs B., Stahel P.F., Gebhard F., Huber-Lang M.S., Perl M. (2013). Inflammatory and apoptotic alterations in serum and injured tissue after experimental polytrauma in mice: Distinct early response compared with single trauma or “double-hit” injury. J. Trauma Acute Care Surg..

[7] Bastian O.W., Kuijer A., Koenderman L., Stellato R.K., van Solinge W.W., Leenen L.P., Blokhuis T.J. (2016). Impaired bone healing in multitrauma patients is associated with altered leukocyte kinetics after major trauma. J. Inflamm. Res..

[8] Keel M., Trentz O. (2005). Pathophysiology of polytrauma. Injury.

[9] Gentile L.F., Cuenca A.G., Efron P.A., Ang D., McKinley B.A., Moldawer L.L., Moore F.A. (2012). Persistent inflammation and immunosuppression: A common syndrome and new horizon for surgical intensive care. J. Trauma Acute Care Surg..

[10] Heffernan D.S., Monaghan S.F., Thakkar R.K., Machan J.T., Cioffi W.G., Ayala A. (2012). Failure to normalize lymphopenia following trauma is associated with increased mortality, independent of the leukocytosis pattern. Crit. Care.

[11] Relja B., Land W.G. (2020). Damage-associated molecular patterns in trauma. Eur. J. Trauma Emerg. Surg..

[12] Sun S., Sursal T., Adibnia Y., Zhao C., Zheng Y., Li H., Otterbein L.E., Hauser C.J., Itagaki K. (2013). Mitochondrial DAMPs increase endothelial permeability through neutrophil dependent and independent pathways. PLoS ONE.

[13] Shao Y., Nanayakkara G., Cheng J., Cueto R., Yang W.Y., Park J.-Y., Wang H., Yang X. (2018). Lysophospholipids and their receptors serve as conditional DAMPs and DAMP receptors in tissue oxidative and inflammatory injury. Antioxid. Redox Signal..

[14] Cohen M.J., Brohi K., Calfee C.S., Rahn P., Chesebro B.B., Christiaans S.C., Carles M., Howard M., Pittet J.-F. (2009). Early release of high mobility group box nuclear protein 1 after severe trauma in humans: Role of injury severity and tissue hypoperfusion. Crit. Care.

[15] Ottestad W., Rognes I.N., Pischke S.E., Mollnes T.E., Andersson U., Eken T. (2019). Biphasic release of the Alarmin high mobility group box 1 protein early after trauma predicts poor clinical outcome. Crit. Care Med..

[16] Mangum L.H., Avila J.J., Hurtgen B.J., Lofgren A.L., Wenke J.C. (2019). Burn and thoracic trauma alters fracture healing, systemic inflammation, and leukocyte kinetics in a rat model of polytrauma. J. Orthop. Surg. Res..

[17] Horst K., Hildebrand F., Pfeifer R., Hübenthal S., Almahmoud K., Sassen M., Steinfeldt T., Wulf H., Ruchholtz S., Pape H. (2016). Impact of haemorrhagic shock intensity on the dynamic of alarmins release in porcine poly-trauma animal model. Eur. J. Trauma Emerg. Surg..

[18] Yang H., Wang H., Andersson U. (2020). Targeting inflammation driven by HMGB1. Front. Immunol..

[19] Yang W.S., Kim J.J., Lee M.J., Lee E.K., Park S.-K. (2018). Ectodomain shedding of RAGE and TLR4 as a negative feedback regulation in high-mobility group box 1-activated aortic endothelial cells. Cell. Physiol. Biochem..

[20] Ruan X., Darwiche S.S., Cai C., Scott M.J., Pape H.-C., Billiar T.R. (2015). Anti-HMGB1 monoclonal antibody ameliorates immunosuppression after peripheral tissue trauma: Attenuated T-lymphocyte response and increased splenic CD11b^+^ Gr-1^+^ myeloid-derived suppressor cells require HMGB1. Mediat. Inflamm..

[21] Huang L.-F., Yao Y.-M., Zhang L.-T., Dong N., Yu Y., Sheng Z.-Y. (2009). The effect of high-mobility group box 1 protein on activity of regulatory T cells after thermal injury in rats. Shock.

[22] Miller A.C., Rashid R.M., Elamin E.M. (2007). The “T” in trauma: The helper T-cell response and the role of immunomodulation in trauma and burn patients. J. Trauma Acute Care Surg..

[23] Percie du Sert N., Hurst V., Ahluwalia A., Alam S., Avey M.T., Baker M., Browne W.J., Clark A., Cuthill I.C., Dirnagl U. (2020). The ARRIVE guidelines 2.0: Updated guidelines for reporting animal research. J. Cereb. Blood Flow Metab..

[24] Darlington D.N., Gonzales M.D., Craig T., Dubick M.A., Cap A.P., Schwacha M.G. (2015). Trauma-induced coagulopathy is associated with a complex inflammatory response in the rat. Shock.

[25] Schneider C.P., Schwacha M.G., Chaudry I.H. (2004). The role of interleukin-10 in the regulation of the systemic inflammatory response following trauma-hemorrhage. Biochim. Biophys. Acta BBA Mol. Basis Dis..

[26] Novotny A.R., Reim D., Assfalg V., Altmayr F., Friess H.M., Emmanuel K., Holzmann B. (2012). Mixed antagonist response and sepsis severity-dependent dysbalance of pro-and anti-inflammatory responses at the onset of postoperative sepsis. Immunobiology.

[27] Kobayashi Y., Kubo A., Iwano M., Sakaguchi Y., Samejima K., Kyoda Y., Yonemasu K., Hashimoto T. (2002). Levels of MCP-1 and GM-CSF mRNA correlated with inflammatory cytokines mRNA levels in experimental autoimmune myocarditis in rats. Autoimmunity.

[28] Heizmann O., Koeller M., Muhr G., Oertli D., Schinkel C. (2008). Th1-and Th2-type cytokines in plasma after major trauma. J. Trauma Acute Care Surg..

[29] Zhao G.-j., Yao Y.-m., Lu Z.-q., Hong G.-l., Zhu X.-m., Wu Y., Wang D.-w., Dong N., Yu Y., Sheng Z.-y. (2012). Up-regulation of mitofusin-2 protects CD4^+^ T cells from HMGB1-mediated immune dysfunction partly through Ca^2+^-NFAT signaling pathway. Cytokine.

[30] Wild C.A., Bergmann C., Fritz G., Schuler P., Hoffmann T.K., Lotfi R., Westendorf A., Brandau S., Lang S. (2012). HMGB1 conveys immunosuppressive characteristics on regulatory and conventional T cells. Int. Immunol..

[31] Parker K.H., Sinha P., Horn L.A., Clements V.K., Yang H., Li J., Tracey K.J., Ostrand-Rosenberg S. (2014). HMGB1 enhances immune suppression by facilitating the differentiation and suppressive activity of myeloid-derived suppressor cells. Cancer Res..

[32] Kerner T., Ahlers O., Spielmann S., Keh D., Bührer C., Gerlach M., Höfler S., Gerlach H. (1999). L-selectin in trauma patients: A marker for organ dysfunction and outcome?. Eur. J. Clin. Investig..

[33] D’Alessio F.R., Kurzhagen J.T., Rabb H. (2019). Reparative T lymphocytes in organ injury. J. Clin. Investig..

[34] Zhang Y., Li X.F., Wu W., Chen Y. (2015). Dynamic changes of circulating T-helper cell subsets following severe thoracic trauma. Int. J. Clin. Exp. Med..

[35] Gupta D.L., Sinha T., Bhoi S., Rao D. (2020). Cytokine Gene Polymorphism and Sepsis. Infectious Process and Sepsis.

[36] Zedler S., Faist E., Ostermeier B., Donnersmarck G.H.V., Schildberg F.-W. (1997). Postburn Constitutional Changes in T-cell Reactivity Occur in CD8^+^ Rather than in CD4^+^ Cells. J. Trauma Acute Care Surg..

[37] Zedler S., Bone R.C., Baue A.E., von Donnersmarck G.H., Faist E. (1999). T-cell reactivity and its predictive role in immunosuppression after burns. Crit. Care Med..

[38] Binkowska A.M., Michalak G., Pilip S., Kopacz M., Słotwiński R. (2018). The diagnostic value of early cytokine response in patients after major trauma–preliminary report. Cent. Eur. J. Immunol..

[39] Wang S., Gao X., Shen G., Wang W., Li J., Zhao J., Wei Y.-Q., Edwards C.K. (2016). Interleukin-10 deficiency impairs regulatory T cell-derived neuropilin-1 functions and promotes Th1 and Th17 immunity. Sci. Rep..

[40] Wood J.J., Rodrick M.L., O’Mahony J.B., Palder S.B., Saporoschetz I., D’Eon P., Mannick J.A. (1984). Inadequate interleukin 2 production. A fundamental immunological deficiency in patients with major burns. Ann. Surg..

[41] Svoboda P., Kantorová I., Ochmann J. (1994). Dynamics of interleukin 1, 2, and 6 and tumor necrosis factor alpha in multiple trauma patients. J. Trauma.

[42] Reinke S., Geissler S., Taylor W.R., Schmidt-Bleek K., Juelke K., Schwachmeyer V., Dahne M., Hartwig T., Akyüz L., Meisel C. (2013). Terminally differentiated CD8^+^ T cells negatively affect bone regeneration in humans. Sci. Transl. Med..

